# Department of Therapeutics

**Published:** 1893-04

**Authors:** David Cerna

**Affiliations:** Galveston; Demonstrator of Physiology in the Medical Dpartment of the University of Texas, etc.


### Abstracts of Current Medical Literature.

### DEPARTMENT OF THERAPEUTICS.

Under the charge of David Cerna, M. D. PH. D., Demonstrator of Physiology in the Medical Dpartment of the University of Texas, etc.

### DUBOISINE SULPHATE IN MENTAL DISORDERS

The sulphate of duboisine recommended by Belmondo sometime ago in the treatment of mental disease, has been further tried with the same excellent results, by Mazzocchi and Antonio. (*Rifoama Medica*, November 15, 1892). Thirty patients were treated, their symptoms varying from a slight mental excitement to violent mania. The drug was given hypodermatically in from $\frac{1}{120}$ to $\frac{1}{32}$ of a grain (0.002 gramme) in the course of the twenty-four hours. The administration of the drug was followed in the majority of cases by deep sleep, this coming on about twenty minutes after the injection and lasting about five hours. No untoward effects was produced by the drug, although there were noticed in almost every case mydriasis, a sense of general weakness, accompanied by a diminution of the pulse-rate, all this however, wearing off on the appearance of sleep. Improvement in the mental condition was observed in the majority of the cases; in a few little benefit was obtained, and in only one case did the drug absolutely fail. Control experiments showed that hypnotic suggestion had nothing in common with the effects of the alkaloid; and experiments with atrophine and morphine, for purposes of comparison, also demonstrated the fact that duboisine is superior to these latter drugs in the treatment under consideration. One patient received, during two months, as many as 50 $\frac{1}{64}$ of a grain (0.001) doses and yet no increase in the amount was found to be necessary. The fear, therefore, of establishing a tolerance for duboisine, need not be entertained.

---

### PHENOCOLL IN MALARIA.

From a study of the uses of phenocoll in the treatment of malarial disease, Dall 'Olio (*Gazette degli Ospistali*, January 14, 1893) draws the following conclusion: 1. The drug does not appear to have potent antipyretic properties as regards fever in general, but it is at least as effective as quinine in the malarial state. 2. Whereas quinine, in a great many instances, gives rise to toxic symptoms, such as ringing in the ears and cutaneous eruptions, phenocoll has not been found to give rise to any such unpleasant effects. 3. Phenocoll succeeds in a certain number of cases in which quinine absolutely fails, and this is an important discovery if only from the point of view that at any time there might arise a difficulty in obtaining a supply of quinine equal to the demand, whereas phenocoll is producible in any quantity. 4. The taste of the drug can easily be masked by means of syrup, and is not objected to even by children.

---

### NAPHTHOL AS A VERMIFUGE.

Naphthol having recently been recommended as a vermifuge, Dubois, of Amiens, has reported a case in which he found it successful after all other vermifuges had failed. The patient was a girl of sixteen who had suffered from vomiting for several months, and the diagnosis pointed to worms as the cause; the vomiting continuing in spite of varied treatment, and the girl becoming much emaciated, he decided to order four grains naphthol three times a day. In the course of a few days she passed thirty-four round worms; the vomiting then ceased and the recovery was rapid.—*London Lancet*, February 18, 1893.

### ZINC CHLORIDE IN THE TREATMENT OF PULMONARY TUBERCULOSIS.

Good effect in the treatment of early pulmonary tuberculosis, by injections of chloride of zinc, has been reported by Jules Comby (*L 'Union Medicale*, No. 1, 1893). In three cases the results were satisfactory, and the author believes that the drug can be injected into the lungs without any special danger. According to his observations the chloride of zinc tends to promote the formation of fibrous tissue, and thus a cure is produced in the same manner as occurs in the natural arrest of the disease. In the cases treated by Comby the disease was confined to the apices. He employed the drug in solution of the strength of 1 in 50 to 1 in 20. Three drops of the solution were injected subcutaneously, the dose being repeated every third or fourth day until six injections had been given. No untoward local or constitutional symptoms were produced by the novel medicament.

---

### THE PHYSIOLOGICAL ACTION OF CERBERINE.

In a recent thesis, (*Wratch* No. 31, 1892—*Gener de Therap*, February 15, 1893) has studied the action of cerberine on frogs, rabbits, dogs, cats and hedgehogs. The substance was administered by the stomach, subcutaneously and intravenously. The effects of cerberine were found to be analogous to those produced by the digitalis group of remedies. The conclusions of the author are as follows; 1. Introduced under the skin, cerberine rarely produces abscesses; neither did it cause inflammation of the conjunctiva. The substance in its action resembles digitaline and digitaleine more than digitoxine. 2. Small doses, 15 hundredths of a milligramme per kilogramme of the body-weight, in dogs, of cerberine, only produced a sense of slight fatigue; in amounts 18 hundredths of a milligramme per killogram of the body-weight, in the same animals, cerberine caused salivation, vomiting, diarrhoea, muscular weakness and dyspnoea. 3. The paralysis which is often produced is thought to be dependent upon an action on the striated muscular fibre. 4. In small quantities, the pulse becomes strong and slow. In large doses the pulse is at first strong and slow, but soon becomes feeble, weak and rapid. In cold-blooded animals, the slowness of the pulse is due to an action of cerberine upon the cardiac muscle; in mammals, to excitation of the pneumogastric nerve. Death is caused by paralysis of the vagi; the left ventricle of the heart is arrested in systole, that of the right side is diastole. 5. The arterial pressure rises slowly, it remains elevated for sometime, then falls slowly or rapidly. 6. The calibre of the vessels is diminished, except those of the kidneys. 7. Cerberine does not seem to influence the central vaso-mortor system; consciousness is preserved; and the vomiting may depend upon an excitation of the vomiting centre in the medulla oblongata. 8. Cerberine is eliminated by the kidneys. 9. Different animals are affected differently by cerberine. Thus, upon rabbits and hedgehogs cerberine does not influence the digestive tract, whereas upon cats and dogs it causes vomiting and diarrrhœa. Dogs are the most susceptible animals to the action of cerberine; to them this substance is fatal in doses of 18 hundredths of a milligramme per killogramme of the body-weight; to cats, in quantities of 31 hundredths of a milligramme per killogramme; to rabbits, 50 milligrammes per killogramme; and the hedgehogs, 120 milligrammes per killogramme.

---

(*Note.*—The technical name and the chemical formula of *analgen*, as they appeared in the February issue of the Journal, were wrong. The name of the drug is *Ortho-oxyethyl-anamoacetyle-amido-chinoline*, its formulabeing $C_{26}$ $H_{14}$ $N_2$ $O_4$.—*D. Cerna.*

